# Using 3D Bioprinted Autologous Minimally Manipulated Homologous Adipose Tissue for Limb Salvage in Treating Diabetic Foot Ulcer

**DOI:** 10.1055/a-2263-7957

**Published:** 2024-04-10

**Authors:** Hyeon Min Yoon, Woo Jin Song

**Affiliations:** 1 Department of Plastic and Reconstructive Surgery, Soonchunhyang University College of Medicine, Seoul, Korea

**Keywords:** adipose tissue, bioprinting, diabetic foot, limb salvage

## Abstract

Reconstructive surgeons face challenges when considering limb salvage methods for the treatment of diabetic foot ulcers (DFUs). In this article, we present our experience with autologous fat grafting as a viable alternative in cases where flap reconstruction is difficult. We encountered a 78-year-old female patient with a nonhealing DFU who had multiple comorbidities, including renal failure and severe peripheral arterial disease. During the initial multidisciplinary meeting, due to extensive necrosis and osteomyelitis, amputation was recommended. However, the patient expressed a strong preference for a salvage procedure and refused amputation. After careful consideration, we opted to reconstruct the patient's foot using three-dimensional bioprinted autologous minimally manipulated homologous adipose tissue. The AMHAT was engrafted well without complications such as autolysis, graft failure, or infection. After the operation, the large defect with partial bone exposure was covered with healthy granulation tissue. The size of the wound decreased to less than half its original size after 6 weeks of surgery, and it decreased to less than 25% after 12 weeks of surgery. The AMHAT may be an appealing treatment option for diabetic foot patients who are unsuitable for flap reconstruction due to comorbidities.

## Introduction

Preserving lower extremity function is a primary objective for physicians treating patients with diabetic foot ulcers (DFUs). 1 Reconstructive surgeons consider various reconstructive options to salvage limbs. Flap reconstruction often yields optimal outcomes; however, its application can be challenging in patients with severe peripheral artery disease (PAD) or those unable to tolerate lengthy operations due to multiple comorbidities. 2 Consequently, these patients frequently undergo minor or major amputations, resulting in a significant decline in their quality of life. Furthermore, long-term mortality rates are substantially high among individuals who have undergone lower extremity amputations, particularly when accompanied by risk factors such as renal disease, diabetes, and PAD. 3 4 Therefore, there is a necessity to develop novel strategies for limb salvage in patients with DFUs.

Autologous fat grafting can be a promising candidate for new treatment options. It is a frequently performed procedure in plastic surgery, serving multiple purposes such as body contour correction and rejuvenation. 5 It is popular due to its advantages, including less invasiveness, easy accessibility, and less donor site morbidity. 6 Recently, there has been an increase in studies demonstrating the potential of autologous fat grafting in promoting wound healing. 7 This increasing interest has led to the development of innovative approaches. Three-dimensional bioprinted autologous minimally manipulated homologous adipose tissue (3D-AMHAT) is a newly developed treatment combining autologous fat grafting and 3D printing technique. 8 9 10 11 In this article, we present our experience in utilizing 3D-AMHAT for limb salvage in patients who were considered challenging candidates for conventional reconstructive surgery.

## Idea

**Fig. 1:**
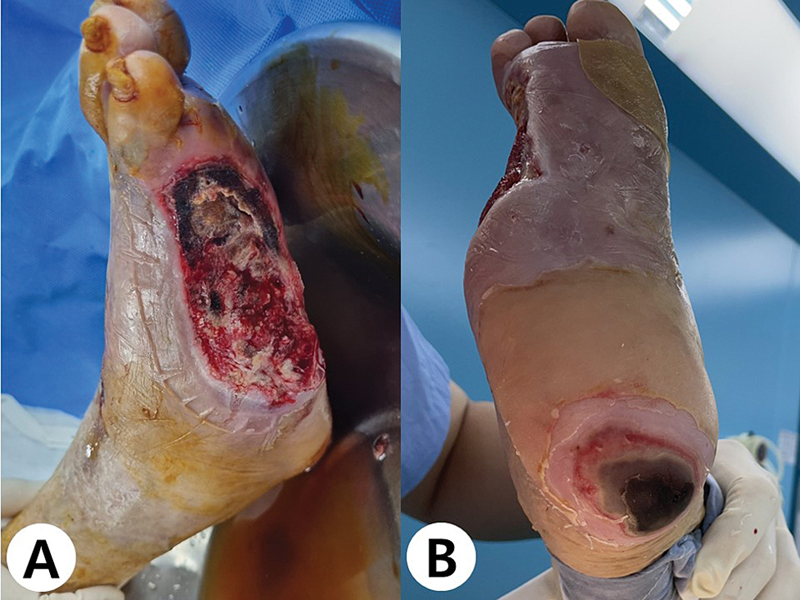
Preoperative photograph of patient's right foot showing large defect on the lateral aspect ( **A** ) and necrosis on the heel ( **B** ).

A 78-year-old female patient presented to our hospital with complaints of right foot pain. She had a medical history of type 2 diabetes mellitus, end-stage renal disease, and PAD. Upon examination, an eschar measuring approximately 1 cm in diameter was observed in the region of the fifth metatarsal bone on her right foot. Lower extremity vascular evaluation revealed an ankle brachial index of approximately 0.5, indicating severe stenosis below the right ankle. The patient underwent percutaneous transluminal angioplasty and received regular follow-up; however, her condition progressively worsened, resulting in deepening ulcer and the detection of concomitant osteomyelitis on radiologic examination. To prevent the further progression of osteomyelitis, treatment of the already necrotic areas was necessary. As a result, she underwent a minor amputation involving parts of the fourth and fifth metatarsal bones. Despite the amputation, the patient's diabetic foot did not exhibit significant improvement, and she developed additional lesion on the heel ( Fig. 1 ). The patient and her family expressed a strong desire to preserve her limb function to the greatest extent possible. During the multidisciplinary meeting, considering her poor vascular condition, age, comorbidities, and the presence of an ongoing DFU on the heel, we recommended amputating her foot. However, the patient and her family declined the amputation and requested an alternative treatment option. Subsequently, we decided to reconstruct her foot using 3D-AMHAT, conservatively resecting the necrotic tissue and administering antibiotics to suppress osteomyelitis.

**Fig. 2:**
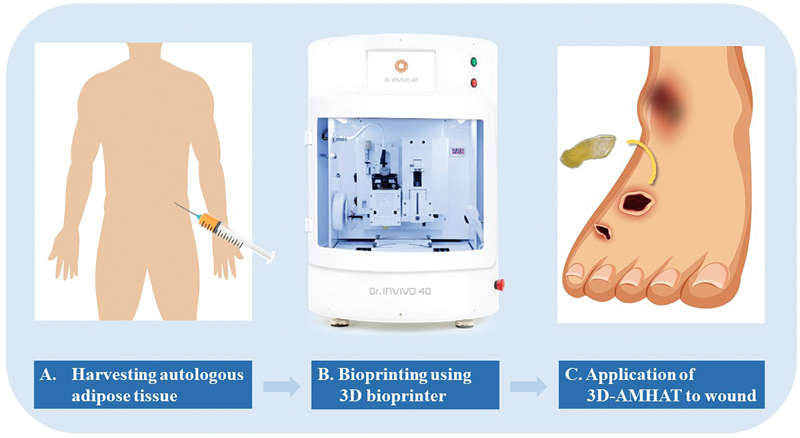
A diagram showing the flow of procedure of 3D-AMHAT. 3D-AMHAT, three-dimensional bioprinted autologous minimally manipulated homologous adipose tissue.

Under general anesthesia, standard liposuction techniques were employed to harvest fat from the patient's abdomen. The lipoaspirate was then centrifuged for 5 minutes at 3,000 rpm, and the second layer was collected into a syringe. The patient's wound was captured and converted into three-dimensional image files using NewCreatorK software (ROKIT Healthcare, Seoul, Korea). According to these files, the 3D bioprinter first printed a scaffold, which was later separated from the patch. Subsequently, a syringe containing lipoaspirates and another filled with fibrin glue (Tisseel; Baxter AG, Vienna, Austria) were inserted into the 3D bioprinter. The lipoaspirates and fibrin glue were then printed in the scaffold in sequence. After hardening, the printed AMHAT was applied to the defect, and a nonadherent dressing, such as Mepitel (Mölnlycke Health Care AB, Gothenburg, Sweden), was placed over it. Subsequently, a secondary dressing comprising foam dressing and cast padding was applied ( Figs. 2 3 4 ).

**Fig. 3:**
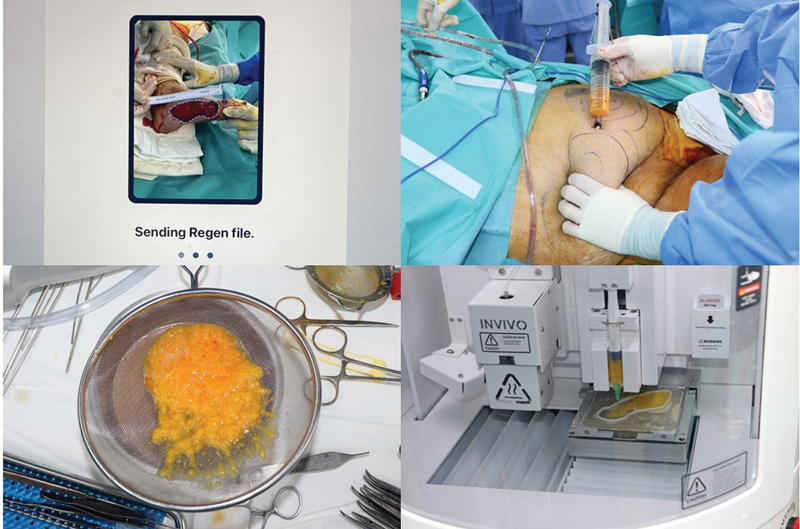
Intraoperative photograph showing autologous fat being harvested by liposuction and printed by a 3D printing machine.

**Fig. 4:**
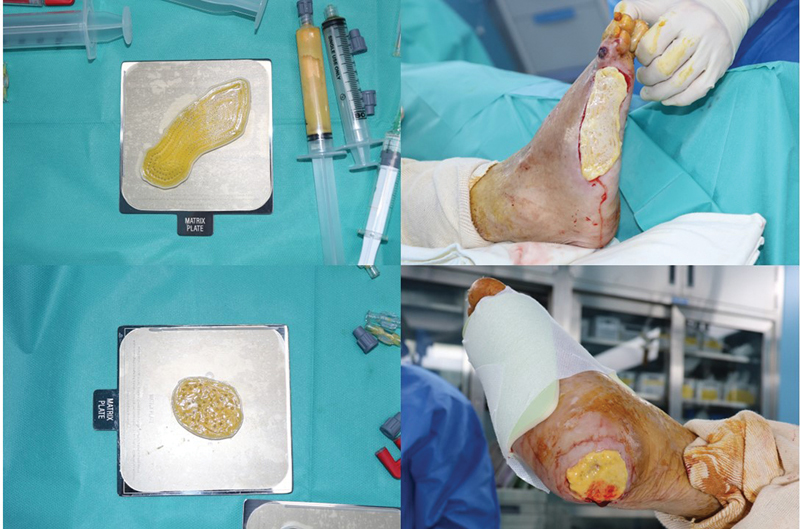
Intraoperative photograph showing printed 3D-AMHAT patch being grafted to patient's defect. 3D-AMHAT, three-dimensional bioprinted autologous minimally manipulated homologous adipose tissue.

**Fig. 5:**
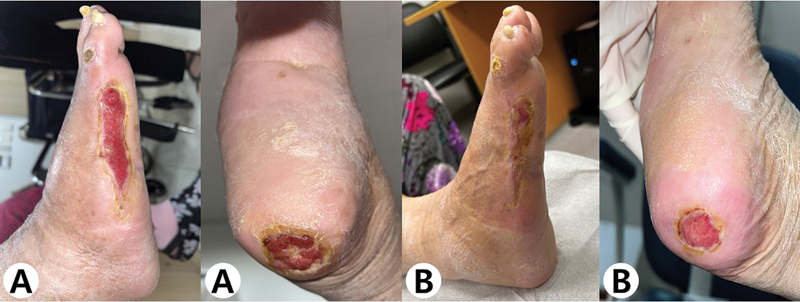
Postoperative photograph after 6 weeks ( **A** ) and 12 weeks ( **B** ). The size of the wound reduced to less than half after 6 weeks after surgery, and complete epithelization was observed on the lateral aspect of the foot 12 weeks after surgery without additional treatment.

The patient's wounds were assessed on a weekly basis, and she was discharged approximately 2 weeks after the surgery. The AMHAT graft was well attached and successfully integrated without any complications such as autolysis, graft failure, or infection. The previously large defect with exposed bone showed healthy granulation tissue coverage. At the 6-week postoperative follow-up visit, a reduction in the wound size by half was confirmed. The wound on the lateral aspect of the foot have completely epithelialized, and the wound on the heel has decreased in size by approximately 75% ( Fig. 5 ).

## Discussion

Debridement plays a crucial role in the treatment of DFUs, particularly when necrotic tissue is present. Following debridement, additional reconstructive measures such as flaps or skin grafts are often necessary for wound closure. 12 However, reconstruction may not always be feasible, particularly in patients with multiple comorbidities and extensive defects. In cases where reconstruction is not possible, amputation may be considered to prevent further deterioration of the condition. But it is important to note that amputation, especially in high-risk patients, can have a significant impact on the patient's overall health and decrease their life expectancy. 13 Therefore, there is a need for alternative reconstructive options that can help avoid major amputations, which significantly impact the patient's quality of life and overall morbidity. Therefore, we propose fat grafting as a promising and innovative option for addressing these challenges.

Fat grafting has been widely used in various fields of plastic surgery, but it has traditionally been considered difficult to reconstruct wounds with raw surfaces using fat grafts alone. Additionally, fat grafting has not been commonly utilized for the treatment of chronic wounds. The mechanism by which fat grafts integrate into the wound site has not been fully elucidated. However, emerging studies suggest that fat grafts promote wound healing and exert a vasogenic effect. 13 14 15 16 Lipoaspirate, which is obtained during fat grafting, contains cells such as preadipocytes and multipotent adipose-derived stem cells. These cells contribute to a vasogenic effect in hypoxic conditions, thereby facilitating wound healing. 4 5 Previously, fat grafts lacked a regular shape, making it challenging to cover irregularly shaped soft tissue defects. However, advancements in 3D printing technology and scaffold materials have made it possible to create fat grafts with precise and regular shapes. Moreover, the use of 3D scanning allows accurate replication of irregular wound shapes, enabling the application of fat grafts to such wounds. The relative simplicity and shorter operative time of fat grafting make it a suitable option for patients at high risk for surgery, and the procedure can be performed under local anesthesia.

There have been few prior studies on the use of 3D-AMHAT for treating DFUs, and none have specifically addressed the treatment of multiple DFUs in distinct vascular territories. 8 9 10 11 Our case demonstrates successful reconstruction using 3D-AMHAT in a high-risk patient with multiple DFUs. However, there are some limitations to our study. Firstly, the complete epithelialization time of approximately 12 weeks in our case differs slightly from previous studies, which reported complete epithelialization within 6 to 8 weeks. However, this discrepancy is likely attributed to statistical bias due to considerations of the patient's age, underlying diseases, and the fact that the treatment was performed in a single case. Additionally, we could not exclude the possibility that the patient's fat cells had a relatively diminished healing potential due to the patient's old age. Secondly, there is limited understanding of the action of fibrin glue, which may potentially aid in wound healing. Further research is needed to clarify the influence of fibrin glue in this regard.

In our case, the patient's preference for a quick return to daily life and reluctance towards additional surgery resulted in discharge with a plan for secondary wound healing. However, it is anticipated that the epithelialization time could be further reduced by combining 3D-AMHAT with skin grafting in patients seeking expedited treatment. The significance of this case lies in the successful salvage of the limb using a fat graft in a patient with multiple DFUs. This suggests that reconstruction surgeons should consider fat grafting as a valuable option for DFU treatment.
